# Chiral Supramolecular Hydrogels Regulating Both Osteoblastogenesis and Osteoclastogenesis

**DOI:** 10.3390/gels11020112

**Published:** 2025-02-05

**Authors:** Beibei Wu, Xiaoqiu Dou, Sravan Baddi, Fengli Gao, Changli Zhao, Chuanliang Feng

**Affiliations:** State Key Lab of Metal Matrix Composites, School of Materials Science and Engineering, Shanghai Key Laboratory for Molecular Engineering of Chiral Drugs, Shanghai Jiao Tong University, 800 Dongchuan Road, Shanghai 200230, China

**Keywords:** chiral supramolecular hydrogel, osteoblastogenesis, osteoclastogenesis

## Abstract

Osteoporosis, a chronic bone disorder, poses a global threat to the health of millions of individuals. The disruption of bone homeostasis is the fundamental cause of osteoporosis. Currently, clinical drugs are employed to promote bone formation via enhancing osteogenesis and/or reduce bone loss via inhibiting osteoclastogenesis. However, it is difficult for the current drugs to simultaneously address the osteoblastogenesis and osteoclastogenesis issues associated with osteoporosis. Hence, L/D-phenylalanine derivatives (L/DPF), combined with Mg^2+^ ions, are employed to assemble into chiral supramolecular hydrogels which facilitate osteocyte activity and inhibit osteoclast function. LPF_Mg hydrogels and DPF_Mg hydrogels demonstrate the opposite supramolecular chirality. Specifically, LPF_Mg hydrogels and DPF_Mg hydrogels are composed of left-handed (M-type) helical nanofibers and right-handed (P-type) helical nanofibers, respectively. The hydrogen bonding and π–π stacking interactions are crucial in the process of hydrogel formation. The chiral left-handed nanofibrous DPF_Mg hydrogels significantly promote osteogenic differentiation of MC3T3 cells and inhibit osteoclast differentiation of RAW267.4 cells, thereby demonstrating substantial potential for applications in improving skeletal health. These findings provide a promising novel perspective on the application of chiral functional materials for osteoporosis therapy.

## 1. Introduction

Osteoporosis is the predominant pathologic condition resulting from excessive bone destruction, and osteoporotic fractures impart catastrophic effects on the health of elderly people [1,2,3,4,5]. This metabolic bone disorder is commonly caused by an imbalance for the homeostasis of the bone tissue, which predisposes to increase fracture risk and fracture severity [6]. Although the commonly used clinical drugs can induce bone formation via enhancing osteoblasts proliferation or prevent bone loss via inhibiting osteoclast differentiation [7,8,9], the efficacy of bone regeneration has still been constrained because these drugs fail to realize improved osteogenesis and inhibit osteoclastogenesis at the same time. Furthermore, these drugs failed to mitigate the imbalance in homeostasis of the bone tissue. Therefore, development of a strategy which allows both enhanced osteoblast activity and restricted osteoclast differentiation is urgently needed for osteoporosis treatment.

Osteoporosis is characterized by a reduction in bone mass and degradation of bone microstructure caused by increased osteoclasts and decreased osteoblasts [10,11]. Hence, both the promotion of osteoblasts generation and the suppression of osteoclasts production play vital roles in osteoporosis therapy, which can alleviate the imbalance in bone homeostasis. For instance, bisphosphonates are recommended as a first-line treatments for post-menopausal osteoporosis as they inhibit the digestion of bone by encouraging osteoclasts to undergo apoptosis, thereby slowing bone loss [12,13]. Parathyroid hormone has been applied to reverse corticosteroid-induced osteoporosis by directly promoting osteoblast proliferation and survival [14,15]. Although this is of great significance in osteoporosis treatment, how to simultaneously facilitate osteoblasts generation and inhibit osteoclasts production remains a challenge [16]. Due to the challenge posed by these clinical drugs in simultaneously promoting osteogenesis and inhibiting osteoclastogenesis, current therapeutic approaches struggle to effectively restore the bone homeostasis. Recent studies have shown that the extracellular matrix (ECM) is closely related to the cellular fate, and modulation of the ECM can regulate osteogenic differentiation of preosteoblasts and osteoclast differentiation of monocyte/macrophage lineage cells [6,17,18]. Attributed to the unique mechanical properties and biocompatibility, functional hydrogel has been widely utilized to mimic the characteristics of the ECM environment that are present in native tissues [19]. Thus, the construction of functional hydrogels to mimic ECM is a promising approach to achieve the simultaneous regulation of osteocytes and osteoclasts.

**Scheme 1:**
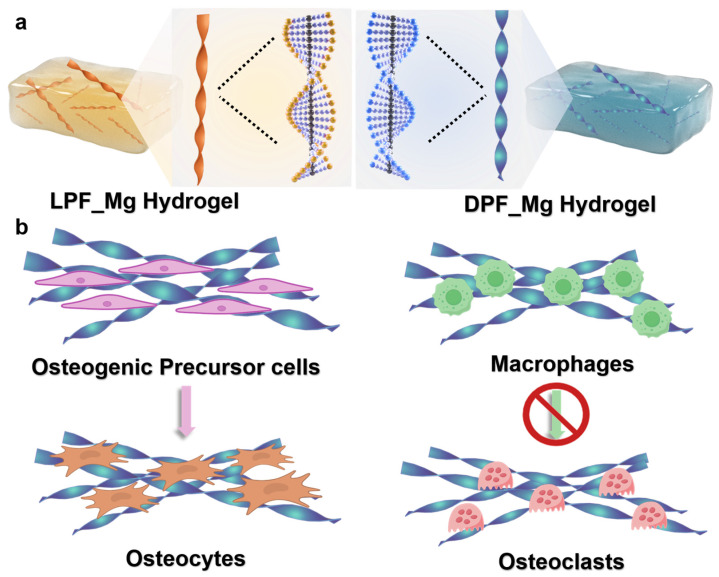
(**a**) A schematic representation of right-handed LPF_Mg and left-handed DPF_Mg hydrogels, respectively. (**b**) DPF_Mg hydrogel constructed by left-handed helical nanofibers promotes osteogenic differentiation and suppresses osteoclastic differentiation.

Herein, we designed and established a chiral supramolecular hydrogel as the ECM to promote osteogenic differentiation and suppress osteoclast differentiation (Scheme 1) [20]. L-phenylalanine derivatives (LPF) combined with Mg^2+^ ions were employed to construct chiral supramolecular hydrogels, which were constructed with right-handed (*P*-type) helical nanofibers. Conversely, D-phenylalanine derivatives (DPF) cooperated with Mg^2+^ ions assembled into the left-handed (*M*-type) nanofibrous hydrogels (Scheme 1a). The structural analysis confirmed that hydrogen bonding and π–π stacking interactions played key roles in the assembled process. Subsequent biological assays first demonstrated that both right-handed LPF_Mg and left-handed DPF_Mg chiral hydrogels exhibited no cytotoxicity for MC3T3 and RAW267.4 cells. Then, it was found that DPF_Mg hydrogels could not only promote osteogenic differentiation of MC3TC cells, but also suppress the osteoclastic differentiation of RAW267.4 cells (Scheme 1b). Compared to the blank control group, DPF_Mg hydrogels enhanced the osteogenic activity by 3.29-fold, whereas the osteoclastogenic activity was reduced by 0.21-fold. These new findings provide an innovative perspective on constructing a chiral supramolecular hydrogel that simultaneously facilitates osteogenic differentiation and suppresses osteoclast differentiation, which may have potential for effective osteoporosis treatment.

## 2. Results and Discussion

### 2.1. Morphological Characterization of L/DPF_Mg Hydrogel

**Figure 1:**
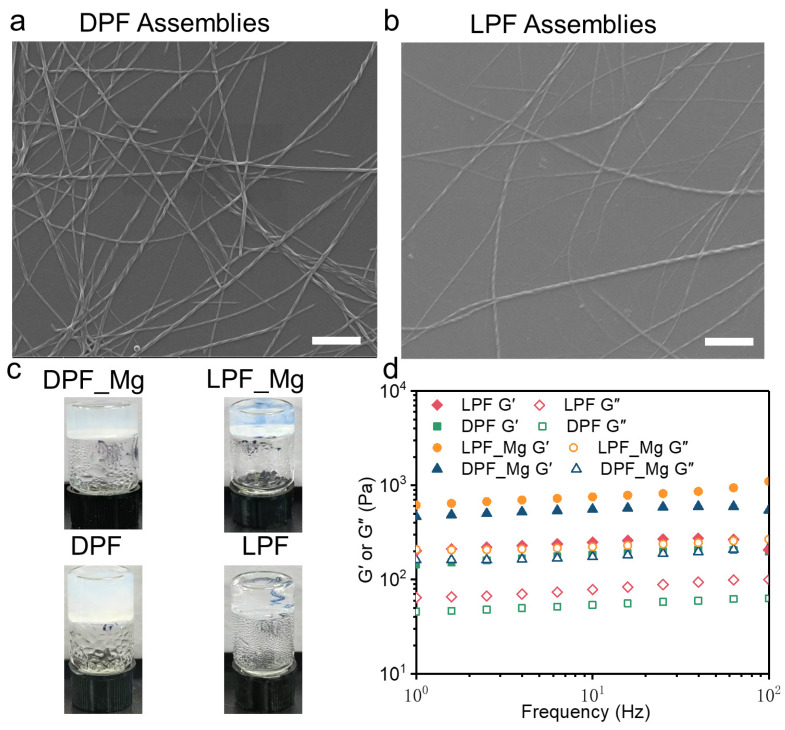
The morphological and mechanical characterizations of L/DPF assemblies. SEM images of (**a**) DPF assemblies and (**b**) LPF assemblies (scale bar = 1 μm). (**c**) Photographs of L/DPF with Mg^2+^ hydrogels and L/DPF hydrogels. (**d**) Rheological properties of L/DPF and L/DPF_Mg hydrogels.

To prepare the chiral supramolecular hydrogel for osteocytes and osteoclast analysis, molecular building blocks, L/D-phenylalanine derivatives (L/DPF), were synthesized according to the previous studies (Figures S1–S3, Supporting Information) [21]. Self-assembled L/DPF hydrogels were driven by heating the solution temperature to 95 °C and cooling it down to room temperature (20 °C). The SEM (Figure 1a,b) images illustrate that L/DPF hydrogels (Figure 1c, 2 mg/mL) were composed of nanofibers. And the LPF and DPF nanofibers demonstrated right-handed (P) and left-handed (M) helical structures, respectively. The L/DPF nanofibers exhibited twisted structures with width ranging from 100 to 200 nm and helical pitches between 150 and 250 nm. L/DPF_Mg hydrogels were prepared by L/DPF molecules mixing with Mg^2+^ ions at a mole ratio of 1:1 in deionized water (Figure 1c). The LPF_Mg and DPF_Mg hydrogels comprised right-handed (*P*) and left-handed (*M*) nanofibers, respectively (Figure S4, Supporting Information).

### 2.2. Mechanical Characterizations of the L/DPF_Mg Hydrogel

To investigate the mechanical properties of the hydrogels, a rheometer was employed to evaluate the prepared L/DPF_Mg supramolecular hydrogel. The dynamic rheological analysis (Figure 1d) indicated that the storage modulus (G′) of L/DPF or L/DPF_Mg samples exceeded the loss modulus (G″), thereby confirming the formation of hydrogels. For LPF_Mg hydrogel, the maximum values of G′ and G″ reached up to approximately 1000 Pa and 250 Pa, respectively. And the maximum values of G′ and G″ for DPF_Mg hydrogel were around 600 Pa and 200 Pa, respectively. However, the maximum values of G′ and G″ for L/DPF hydrogel were only about 200 Pa and 90 Pa. Furthermore, the rheological parameters related to the share rate vs. shear stress was shown on Figure S5, which were found to fit the Herschel–Bulkley model [22]. Hence, the introduction of Mg^2+^ ions significantly increased increase the mechanical properties of L/DPF hydrogels.

### 2.3. Chiral Characterizations of the L/DPF_Mg Hydrogel

**Figure 2:**
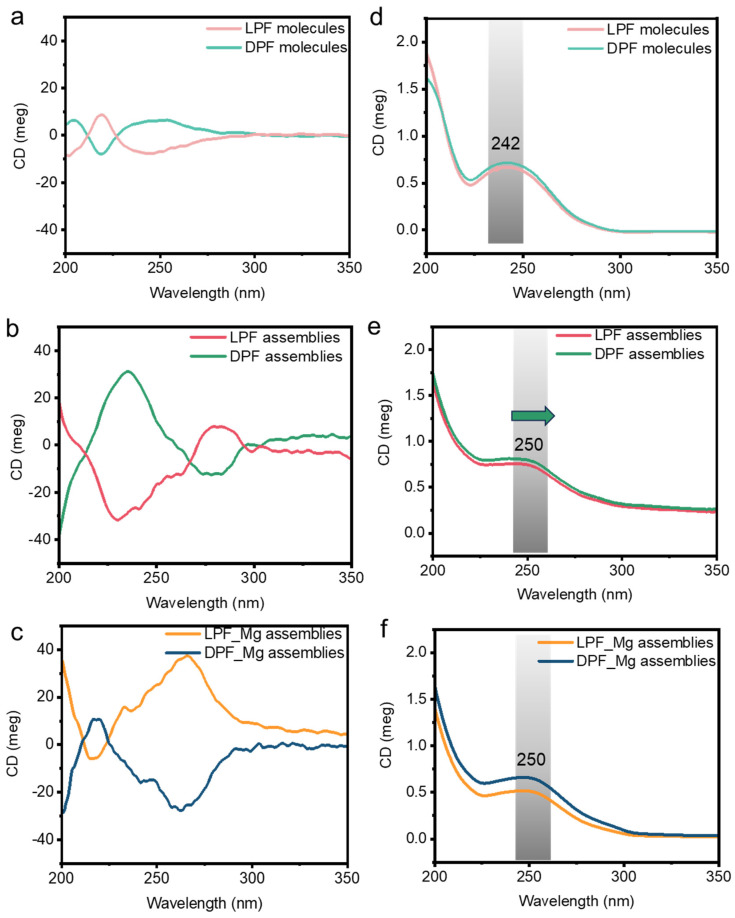
The chiral characterizations of the molecules and hydrogels. (**a**) The CD spectrum and (**d**) UV–Vis spectrum of L/DPF molecules. (**b**) The CD spectrum and (**e**) UV–Vis spectrum of L/DPF hydrogels. (**c**) The CD spectrum and (**f**) UV–Vis spectrum of L/DPF_Mg hydrogels.

The chirality of the formed supramolecular hydrogel was investigated by CD spectroscopy (Figure 2). CD spectra of LPF and DPF enantiomers dissolved in MeOH at a concentration of 2 mg/mL showed Cotton effects at 218 and 242 nm (Figure 2a), which could be attributed to the amide carbonyl groups and phenyl groups of L/DPF chiral molecules, respectively [20]. Compared to the CD signals of LPF molecules, the LPF fibrous hydrogel in H_2_O (2 mg/mL) presented a negative dichroic signal at 223 nm and a positive one at 263 nm, with significantly enhanced intensity due to chiral amplification arising from the asymmetric spatial stacking of LPF chiral molecular building blocks (Figure 2b) [23]. Additionally, the mirror-imaged CD spectrum of DPF assemblies also exhibited positive and negative Cotton effects at 223 nm and 263 nm, respectively. After the addition of Mg^2+^ ions, the intensity of CD signals decreased at 219 nm and increased at 263 nm compared to L/DPF hydrogels (Figure 2c). We hypothesized that the variations in CD signals were attributed to the alterations of the intermolecular forces among L/DPF molecules induced by the addition of Mg^2+^. Compared to the UV–Vis signals of L/DPF molecules (Figure 2d,e), a pronounced red shift was observed in the L/DPF hydrogel, which illustrated that J-type aggregations induced by π–π stacking were present in L/DPF assemblies [24]. Figure 2f shows that there were no significant variations in UV–Vis signals between the L/DPF hydrogel and the L/DPF_Mg hydrogel. Thus, the addition of Mg^2+^ ions had no influence on the L/DPF molecules assembling. These data demonstrated that L/DPF hydrogel exhibited supramolecular chirality, while the introduction of Mg^2+^ ions had little influence on supramolecular chirality.

### 2.4. Spectral Characterizations of the Chiral Supramolcelcuar Hydrogel

**Figure 3:**
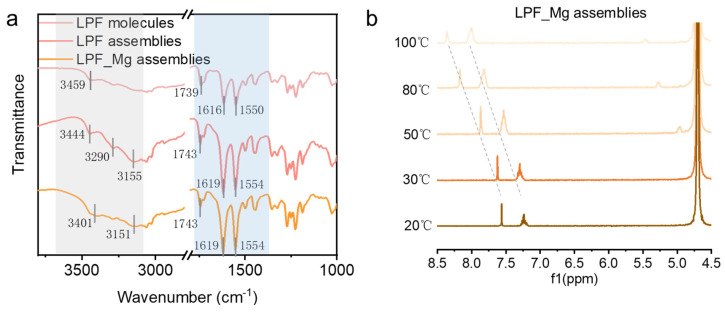
Spectral characterizations of LPF and LPF_Mg assemblies. (**a**) FTIR spectrum of LPF molecules, LPF assemblies and LPF_Mg assemblies. (**b**) ^1^H NMR spectrum of LPF_Mg assemblies at different temperature.

FTIR spectroscopy was employed to further elucidate the intermolecular interactions between L/DPF assemblies and Mg^2+^ ions (here taking LPF as example). The FTIR spectra (Figure 3a) revealed that a red shift in the broad peak centered at 3459 cm^−1^ (−OH stretching vibration of LPF molecules) in the spectrum of LPF assemblies compared to 3444 cm^−1^ of LPF molecules, which indicated the formation of an intermolecular hydrogen bond between the LPF molecules during assembly [25]. And the introduction of Mg^2+^ ions might cause the variation in -OH stretching vibration, resulting in the red shift from 3444 cm^−1^ to 3401 cm^−1^. Another -OH stretching vibration at 3290 cm^−1^ could be observed in the spectrum of LPF assemblies, whereas the peak at 3290 cm^−1^ in the spectrum of LPF molecules and LPF_Mg assemblies was markedly attenuated [26]. These data further confirmed that the assembly of LPF molecules induced the variations in -OH stretching vibration, and that the additions of Mg^2+^ ions could alter the -OH stretching vibration of LPF assemblies. Compared to the LPF molecules, a new broad peak corresponding to N−H bending vibration at 3155 cm^−1^ could be seen in the spectrum of LPF assemblies [27]. And the introduction of Mg^2+^ ions did not have a strong impact on the broad peak at 3151 cm^−1^ of LPF_Mg assemblies. For LPF molecules, the stretching vibration band at 1739 cm^−1^ was attributed to −C=O stretching vibration, while the peaks at 1616 cm^−1^ and 1550 cm^−1^ were attributed to N−H bending vibration [28]. For LPF assemblies and LPF_Mg assemblies, these characteristic peaks (1743 cm^−1^, 1619 cm^−1^ and 1554 cm^−1^) exhibited only slight shifts. Therefore, these data illustrated the formations of intermolecular hydrogen bonds, which were formed between the -OH stretching vibration of LPF molecules in the assemblies, and the introduction of Mg^2+^ also had an impact on the hydrogen bond formed by -OH stretching vibration.

Next, the ^1^H NMR spectra of L/DPF_Mg assemblies at different temperatures was applied to investigate the intermolecular forces (here also taking LPF as example). As shown in Figure 3b, the characteristic peaks at approximately 7.2 ppm and 7.6 ppm were attributed to the aromatic rings of the L/DPF molecules [29]. With increasing temperature, the characteristic peaks of the aromatic rings exhibit an upfield shift from around 7.2 to 7.9 ppm and from 7.6 to 8.3 ppm, respectively. In contrast, the characteristic peaks at 4.7 ppm remained unchanged. Thus, we hypothesized that intermolecular π–π stacking interactions among aromatic rings were crucial in the formation of L/DPF_Mg assemblies.

**Figure 4:**
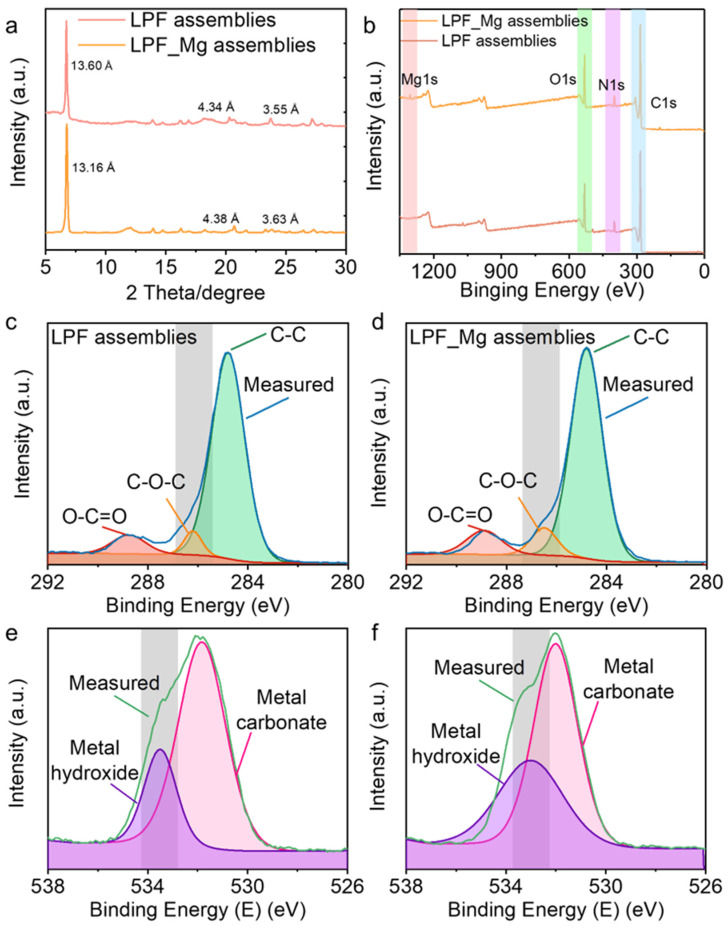
Spectral characterizations of LPF nanofibers. (**a**) XRD spectrum of LPF assemblies and LPF_Mg assemblies. (**b**) XPS survey spectra of LPF assemblies and LPF_Mg assemblies. C 1s high-resolution spectra of (**c**) LPF assemblies and (**d**) LPF_Mg assemblies. O 1s high-resolution spectra of (**e**) LPF assemblies and (**f**) LPF_Mg assemblies.

Subsequent XRD analyses were then performed to elucidate the distinct structural characteristics of the L/DPF and L/DPF_Mg assemblies (here taking LPF as example). As illustrated in Figure 4a, the XRD spectrum of LPF assemblies revealed a characteristic peak at 2θ = 6.50°, corresponding to the d-spacings of 13.60 Å. The most intense peak (13.60 Å) corresponded to the molecular length of LPF assemblies [30]. Additionally, the diffractograms of LPF assemblies revealed an additional diffraction peak, observed at 2θ = 20.45° (the d -spacings was 4.34 Å), which corresponded to the hydrogen bonding within LPF assemblies [31]. And another characteristic diffraction peak at 2θ = 25.06° assigned to the d-spacing of 3.55 nm was observed, indicating the presence of π–π stacking interactions within the assemblies [32]. Following the addition of Mg^2+^ ions, the characteristic peak at 2θ = 6.71° (with the d-spacings of 13.60 Å) corresponding to the molecular length did not change much. However, the diffraction peaks at 2θ = 20.25° (with d-spacings of 4.38 Å) and 2θ = 24.51° (with d-spacings of 3.63 Å) exhibited slight changes. Therefore, hydrogen bonding and π–π stacking interactions played important roles in the assembly process, and the introduction of Mg^2+^ ions might impact the hydrogen bonding and π–π stacking interactions within the assemblies.

Since it was known that the surface chemical composition may impact cellular behavior, the chemical compositions of the LPF assemblies and LPF_Mg assemblies were characterized by XPS. The high-resolution XPS surveys were performed in C 1s, O 1s, N 1s and Mg 1s regions for LPF assemblies and LPF_Mg assemblies. As shown in Figure 4b and Tables S1 and S2 Supporting Information, the characteristic peaks of Mg 1s (1304.24 eV) could be found in the LPF nanofibers after the introduction of Mg^2+^ ions. Furthermore, XPS results revealed that C 1s (~284 eV), N 1s (~532 eV) and O 1s (~399 eV) peaks were present in both LPF and LPF_Mg assemblies (Figure 4b and Tables S1 and S2, Supporting Information) [33,34]. Then, from the full XPS spectra, the characteristic peaks of C 1s and O 1s were observed in these assemblies. Figure 4c,d and Tables S3 and S4 in the Supporting Information show that the XPS spectrum for C 1s could be deconvoluted into the peaks of C-C (284.88 eV, 86.57 atom %), C-O-C (286.50 eV, 6.12 atom %) and O-C=O (288.90 eV, 7.31 atom %). The introduction of Mg^2+^ ions to the LPF assemblies caused slight changes in the peak of C-O-C (286.20 eV, 5.38 atom %) and O-C=O (288.76 eV, 8.15 atom %) [35]. Figure 4e,f and Tables S5 and S6 in the Supporting Information show that the XPS spectrum of O 1s can be deconvoluted into distinct peaks corresponding to metal hydroxide (533.50 eV, 95.48 atom %) and metal carbonate (532.15 eV, 4.52 atom %) [36]. And the introduction of Mg^2+^ ions to the LPF assemblies had a large influence on the peak of metal hydroxide (532.16 eV, 95.48 atom %) and metal carbonate (533.60 eV, 4.52 atom %). Hence, the XPS results demonstrated that the introduction of Mg^2+^ ions had an impact on the interaction of oxygen-containing groups in the molecular stacking for assembling.

### 2.5. Cytotoxicity of the Chiral Supramolecular Hydrogel

**Figure 5:**
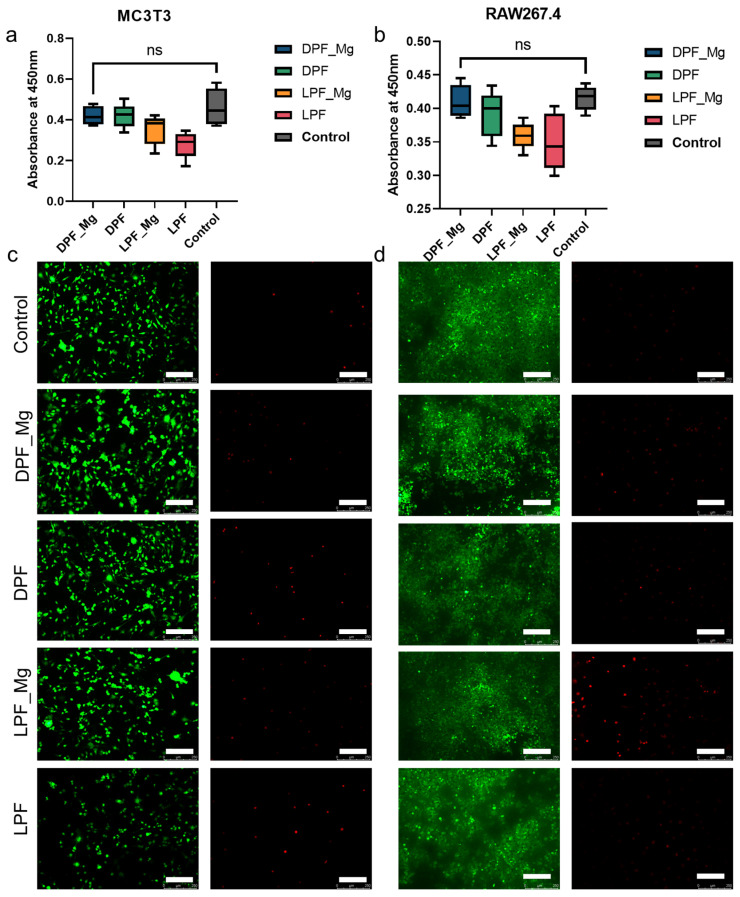
Cytotoxicity of the chiral supramolecular hydrogel. The cell growth-related OD value of (**a**) MC3T3 and (**b**) RAW267.4 cells investigated by CCK8 assays in L/DPF, L/DPF_Mg hydrogels-based ECM and the control group. Live/dead staining of (**c**) MC3T3 and (**d**) RAW267.4 cells in L/DPF, L/DPF_Mg hydrogels-based ECM and the control group. Scale bar = 200 μm. Data were represented as the mean ± SEM via GraphPad Prism with five replicates per data point. (NS, no significance).

In recent years, MC3T3-E1 cells, pre-osteoblast cells from mouse, have generally been applied as a cell source for osteogenic differentiation research [37]. Macrophages served as the precursors of osteoclasts, which were major bone cells associated with bone tissue [38]. To access the cytotoxic effects of L/DPF and L/DPF_Mg hydrogels, cck8 assays were conducted to evaluate the cell growth of MC3T3 and RAW267.4 cells seeding on different nanofibers films as chiral ECM, including cells treated on L/DPF and L/DPF_Mg hydrogels and the blank control group (Figure 5a,b). After MC3T3 and RAW267.4 cells were incubated for 48 h, the OD value related to cell growth in the blank control group were slightly higher than the other groups (MC3T3: 0.46 ± 0.09, RAW267.4: 0.42 ± 0.01). And the OD values related to cell growth in the LPF (MC3T3: 0.28 ± 0.07, RAW267.4: 0.35 ± 0.04) and LPF_Mg (MC3T3: 0.35 ± 0.07, RAW267.4: 0.36 ± 0.02) hydrogels groups were marginally lower than those observed in the DPF (MC3T3: 0.42 ± 0.06, RAW267.4: 0.39 ± 0.03) and DPF_Mg (MC3T3: 0.42 ± 0.05, RAW267.4: 0.41 ± 0.02) hydrogels groups. As shown in Figure 5c,d, live/dead staining images indicated that the quantity of green-stained MC3T3 cells was approximately equivalent across all groups, and the quantity of green-stained RAW267.4 cells was also roughly the same in these groups. However, only a few red-stained cells for MC3T3 and RAW267.4 cells were found in each group. Therefore, there was no obvious cytotoxicity in MC3T3 and RAW267.4 cells seeding on L/DPF and L/DPF_Mg hydrogels as chiral ECM.

### 2.6. Regulation of Osteogenesis and Osteoclastogenesis by Chiral Nanofibers

**Figure 6:**
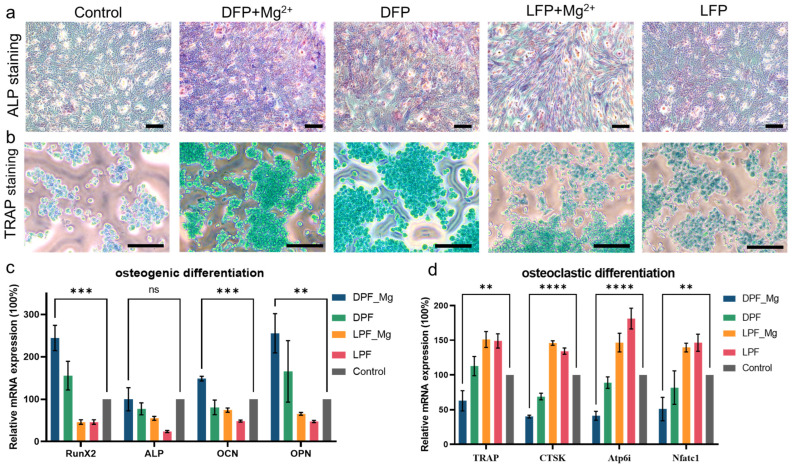
Regulation of osteogenesis and osteoclastogenesis by the chiral supramolecular hydrogel as the ECM. (**a**) ALP staining for MC3T3 cells in L/DPF, L/DPF_Mg and the control group. (**b**) TRAP staining for RAW267.4 in L/DPF, L/DPF_Mg and the control group. Scale bar = 200 μm. RT-qPCR of (**c**) RUNX2/ALP/OCN/OPN genes for MC3T3 cells and (**d**) TRAP/CTSK/Atp6i/Nfate1 genes for RAW 267.4 cells in L/DPF, L/DPF_Mg and the control group. Data were represented as the mean ± SEM via GraphPad Prism with five replicates per data point. (NS, no significance., ** *p* < 0.01, *** *p* < 0.001, **** *p* < 0.0001).

To further investigate how the chiral supramolecular hydrogel influenced osteoporosis therapy, we examined that the osteogenic differentiation of MC3T3 cells and osteoclastic differentiation of RAW267.4 cells seeded on chiral ECM. When MC3T3 cells had reached 80% confluency, osteogenic differentiation was induced with the osteogenic differentiation medium. The alkaline phosphatase (ALP) activity expressed by MC3T3 cells cultured on L/DPF and L/DPF_Mg supramolecular hydrogels as ECM was determined after 14 days of differentiation [39]. Figure 6a showed that osteogenic differentiation (purple staining) of MC3T3 cells on DPF_Mg hydrogel ECM was much stronger than those cells cultured on other nanofibers. Then, to quantitatively assess these observations, we evaluated the ratio related to staining intensity of positively stained cells in DPF_Mg group compared to the control group. The ALP assays’ positive rate for osteogenic differentiation in the DPF_Mg group was approximately 3.29-fold higher than the control group (Figure S6). Subsequently, similar findings were observed for gene expression, tested via RT-qPCR. The relative expression of RUNX2/ALP/OCN/OPN was significantly elevated in MC3T3 cells cultured on DPF_Mg supramolecular hydrogels as the ECM. This finding indicated that the left-handed helical nanofibers based hydrogel promote the expression of the related osteogenic differentiation genes compared to the other groups (Figure 6c). To study the influence of the chiral supramolecular hydrogel on osteoclastic differentiation, we seeded RAW267.4 cells on chiral hydrogel as the ECM, and the osteoclastic differentiation of those cells was induced with M-CSF and RANKL [40]. After 5 days differentiation, TRAP staining was used to investigate the influence of chiral nanofibers on osteoclast differentiation [41]. More TRAP-positive multinucleated cells (staining in purple) were found in LPF, LPF_Mg, and the control group than DPF and DPF_Mg group (Figure 6b). The TRAP assays positive rate for osteoclast differentiation in DPF_Mg group was approximately 0.21 relative to the control group (Figure S6). Additionally, RT-qPCR revealed the significant downregulation of the osteoclastic differentiation-related genes TRAP/CTSK/Atp6i/Nfate1 in the DPF_Mg group compared to the other groups (Figure 6d). These data illustrated that the DPF_Mg hydrogel could promote osteogenic differentiation while inhibiting osteoclastic differentiation.

### 2.7. Disccusion

Osteoporosis is the most common pathological condition resulting from excessive bone destruction [42]. The disruption of bone homeostasis is the primary underlying cause of osteoporosis. The crux of osteoporosis treatment is to develop a strategy which can concurrently promote the generation of osteoblasts and inhibit the production of osteoclasts [43]. In this research, we prepared the chiral supramolecular hydrogel for osteoporosis therapy. Furthermore, since the addition of Mg^2+^ ions is widely applied to improve the osteogenesis of implants in clinics, we incorporated Mg^2+^ ions into the chiral hydrogel. Through the structural analyses, it was determined that the incorporation of Mg^2+^ ions has no significant impact on the supramolecular chirality. However, it was observed that the presence of Mg^2+^ ions could influence oxygen- containing groups during the assembly process.

To investigate the effect of the chiral supramolecular hydrogel on osteoblastogenesis and osteoclastogenesis, MC3T3 cells were chosen as the typical osteogenesis precursor cells and RAW267.4 cells were selected as osteoclast precursors for the osteoporotic research. The ALP and TRAP assays were applied to indicate that the DPF_Mg hydrogel could facilitate osteoblasts generation and inhibit osteoclasts production, compared to other chiral nanofibers-based hydrogels. The osteoblasts generation-related genes were upregulated in the DFP_Mg group, while osteoclasts production-related genes were downregulated in the DPF_Mg group compared to the other groups. We speculated that the observed results were attributed to the left-handed nanofibers-based hydrogel, which may favor cell spreading. For MC3T3 cells, the increased cell spreading area could promote the differentiation of adherent cells [44]. Conversely, for RAW267.4, a larger cell spreading area might impede non-adherent cell differentiation [45]. Therefore, the chiral supramolecular hydrogel could alleviate the imbalance of bone homeostasis, which is a promising strategy for osteoporosis therapy.

## 3. Conclusions

In summary, L/D-phenylalanine derivatives (L/DPF) combined with Mg^2+^ ions were employed to develop the chiral supramolecular hydrogel for regulating both osteoblastogenesis and osteoclastogenesis. LPF_Mg hydrogels consisted of left-handed (M-type) helical nanofibers, whereas DPF_Mg hydrogels were composed of right-handed (P-type) helical nanofibers. Both right-handed LPF_Mg and left-handed DPF_Mg nanofibers-based hydrogels exhibited no cytotoxicity for bone-related cells (MC3T3 and RAW267.4 cells). Notably, DPF_Mg hydrogels not only promoted osteogenic differentiation of MC3T3 cells, but also inhibited the osteoclastic differentiation of RAW267.4 cells, thereby mitigating the imbalance of bone homeostasis. This study broadens the potential applications of the chiral supramolecular hydrogel and presents a promising strategy for balancing the bone homeostasis. Additionally, due to the function related to the bone-associated cells differentiation, the study provides a promising approach for bone-related disorders caused by abnormal skeletal cells differentiation (e.g., osteogenesis imperfecta and femoral head necrosis).

## 4. Materials and Methods

### 4.1. Materials

L/D-phenylalanine methyl ester hydrochloride, 1,4-benzenedicarbonyl dichloride and diglycol were procured from Aladdin Chemicals, Shanghai, China and could be used without further purification. Dulbecco’s Modified Eagle Medium (DMEM), RPMI 1640 medium, 0.25% Trysin-EDTA and phosphate-buffered saline (PBS) were purchased from Gibco, Life Technologies, GrandIsland, USA. The cell counting assay kit (CCK8) was obtained from Dojindo, Kyushu, Japan. The LIVE/DEAD Cell Viability Assays Kit was procured from KeyGEN BioTECH, Nanjing, China. The primers used in RT-qPCR were synthesized by Generay Biotechnology, Shanghai, China.

### 4.2. L/DPF Synthesis

L/D-phenylalanine methyl ester hydrochloride, 1,4-benzenedicarbonyl dichloride and diglycol were used for synthesis of L/DPF molecules, referencing previous studies [20].

### 4.3. Fabrication of Chiral Supramolecular Hydrogel

The magnesium chloride hexahydrate was prepared as a 2 mg/mL solution in deionized water. Next, the synthesized L/DPF were added to the Mg^2+^ ions solution at the L/DPF: Mg^2+^ mole ratio of 1:1. Then, the mixed solution was heated to 95 °C for a couple of minutes until the L/DPF molecules were completely dissolved. After being placed in room temperature overnight, the chiral supramolecular hydrogel was formed.

### 4.4. Morphological Characterization

The scanning electron microscopy (SEM) instrument model and test conditions were presented referring to prior studies. A 20 uL amount of L/DPF and L/DPF_Mg hydrogels was prepared on a silicon wafer and dried in an oven at 40–50 °C overnight. After the samples were coated with an ultrathin gold layer (5 nm), the morphological characterization could be observed with SEM. The measurement applied was 5 kV voltage.

### 4.5. Rheological Characterization

Rheological characterization of the L/DPF and L/DPF_Mg hydrogels was performed by an AR G2 rheometer (TA Instruments, New Castle, DE, USA) equipped with a 20 mm diameter parallel-plate geometry [46]. The experiments were conducted through dynamic frequency sweep measurements over a frequency range of 0.1 to 10 Hz at a controlled temperature of 25 °C. A sinusoidal shear strain of 0.1% was applied to obtain the storage modulus (G′) and loss modulus (G″) data.

### 4.6. Structural Characterizations

The circular dichroic (CD) spectra were recorded on the JASCO J-1500 CD spectrometer(Tokyo, Japan) on a bandwidth of 1.0 nm, covering the wavelength range of 190–400 nm. Both of the test concentration of L/DPF and L/DPF_Mg hydrogel solution in a quartz colorimetric cuvette with 1 mm path length were 0.5 mg/mL. Both the CD signal and the UV–Vis spectrophotometer (UV–Vis) signal could be recorded by the instrument.

Fourier transform infrared (FTIR) spectrometer was conducted using Bruck EQUINOX55 spectrometer (Karlsruhe, Germany). This analysis involved the preparation of a sample by mixing freeze-dried hydrogel powder with a potassium bromide (KBr) pellet. Measurements were taken across a wavelength range from 4000 to 400 cm^−1^.

The surface chemical component was characterized utilizing XPS. The L/DPF_Mg hydrogel was deposited onto a silicon wafer substrate. All samples were placed in an oven at 45 °C under ambient pressure for overnight period. The details regarding the XPS instrument model, silicon wafer specifications and testing parameters were documented in previous studies. The analysis of the XPS data were conducted using CasaXPS software (2.3.16).

X-ray diffraction (XRD) was analysis was conducted using a Rigaku D/Max-2500 X-ray diffractometer (Tokyo, Japan), employing Cu Ka radiation (λ = 1.5406 Å) at a scanning speed of 2° per min. The diffractometer operated under a voltage of 40 kV and a current of 150 mA.

### 4.7. Cell Culture

MC3T3 cells were cultured in DMEM medium supplemented with 10% fetal calf serum (FBS) and 1% penicillin and streptomycin (PS), while RAW267.4 cells were maintained in a RPMI 1640 medium with 10% FBS, 1% penicillin and streptomycin. Cell passaging of the two cells lines was conducted every 3 to 5 days. All cell cultures were incubated at 37 °C in a humidified atmosphere of 5% CO_2_.

### 4.8. Osteogenic Differentiation

MC3T3 cells were initially seeded onto chiral supramolecular hydrogel within 6-well plates at a density of 1 × 10^5^ cells per well and cultured in completed medium (DMEM with 10% FBS and 1% PS). After 24 h incubation, the culture medium was replaced with osteogenic media (Cyagen, Santa Clara, CA, USA) for 14 days.

RAW267.4 cells were also seeded onto chiral supramolecular hydrogel in 6-well plates at a density of 1 × 10^5^ cells per well and cultured in completed medium (1640 with 10% FBS and 1% PS). Following a 12 h incubation, the cell medium was substituted with the completed medium with M-CSF (100 ng /mL) RANKL (100 ng /mL) for 5 days.

### 4.9. LIVE/DEAD Cell Viability Assays

In order to investigate biocompatibility of chiral supramolecular hydrogel, MC3T3 and RAW267.4 cells were stained with LIVE/DEAD Cell Viability Assays Kit (KeyGEN BioTECH, KGAF001). 1 × 10^6^ cells well^−1^ cells were seeding on the chiral supramolecular hydrogel for 1 days. After washing cells with PBS, cells were incubated with (2 μM) Calcein AM and (8 μM) PI for 30–45 min. Subsequently, a fluorescence microscope (Olympus BX51, Tokyo, Japan) was utilized to acquire images for assessing the biocompatibility of nanofibers.

### 4.10. Cell Growth Assays

Cells were seeded on 96-well plates coating with chiral supramolecular hydrogel at a density of 1 × 10^5^ cells well^−1^ during the exponential growth phase. Following a 48 h incubation period, CCK8 reagents were added to each well and incubated for 4 h. Subsequently, a microplate reader (ELX800, Bio Tek, Winooski, VT, USA) was applied to record the optical density at 450 nm with a microplate reader.

### 4.11. Alkaline Phosphatase (ALP) Activity Assay

After a 14-day incubation period of MC3T3 cells in osteogenic differentiation medium, the cells were fixed using 4% PFA at 4 °C for about 30 min. Then, PFA was removed and the MC3T3 cells were rinsed with PBS, ALP activity was assessed with nitro blue tetrazolium (NBT) and a 5-bromo-4-chloro-3-indolyl phosphate (BCIP) (NBT/BCIP) staining kit (Beyotime, Shanghai, China).

### 4.12. Tartrate-Resistant Acid Phosphatase (Trap) Staining

The TRAP staining procedure was conducted by a TRAP Staining Kit (Solarbio, Beijing, China) according with the manufacturer’s protocols. RAW267.4 cells were fixed with the fixative solution. After removing the fixative solution and washing with PBS, the incubation solution was treated with the cells for 45–60 min for 37 °C. Then, methyl green was used as a counterstain.

### 4.13. Quantitative Real-Time PCR

Total RNA was extracted from MC3T3 and RAW267.4 cells cultured on the chiral supramolecular hydrogel and employed by Trizol Reagent (Beyotime, Shanghai, China) according to the manufacturer’s introductions. RNA yield was quantified with a NanoDrop 2000 spectrophotometer (Thermo Scientific, Waltham, MA, USA). The isolated RNA, less than 2 ug, was reverse transcribed into cDNA using the TRUEscript RT MasterMix kit (For real time PCR) (Aidlab Biotechnologies, Beijing, China) for real-time PCR applications. Subsequently, qPCR was conducted with a 2 × Sybr Green qPCR Mix (Aidlab Biotechnologies, Beijing, China) on a LightCyclerˆ@96 System 3 real-time PCR system (Roche, Basel, Switzerland). The relative amount of mRNA was assessed via the 2(-ΔΔCt) method normalized by GAPDH. The description of the primer pairs used in this study was given in Supporting Information Table S7.

### 4.14. Statistical Analysis

Numerical data in the manuscript were represented as the mean ± SD, and analyzed via GraphPad Prism (9.5) and Origin (10.1). The statistical comparison between three or more groups was performed with one-way ANOVA, and the comparison between two groups was conducted using a Student’s *t*-test. Statistical significance was designated as follows: NS, no statistical significance, * *p* < 0.05, ** *p* < 0.01, *** *p* < 0.001.

## Data Availability

The raw data supporting the conclusions of this article will be made available by the authors on request.

## Supplementary Materials

The following supporting information can be downloaded at: https://www.mdpi.com/article/10.3390/gels11020112/s1. Figure S1: Synthesis procedures. Figure S2: ^1^H NMR spectra. Figure S3: Mass spectrum. Figure S4: SEM images. Figure S5: Rheological parameters. Figure S6: Relative ratio. Tables S1–S6: XPS survey. Table S7: Primers.
