# Supplementary material for: Chiral Supramolecular Hydrogels Regulating Both Osteoblastogenesis and Osteoclastogenesis
Source: Gels. 2025 Feb 5;11(2):112. doi: 10.3390/gels11020112 (PMC11855158; doi:10.3390/gels11020112)
Supplement: Supplementary file 1 [file gels-11-00112-s001.zip › gels-3440980-supplementary.pdf]

Suppermentary Informations

Chiral Supramolecular Hydrogels Regulating Both Osteoblastogenesis and Osteoclastogenesis

Beibei Wu, Xiaoqiu Dou*, Sravan Baddi, Fengli Gao, Changli Zhao and Chuanliang Feng*

State Key Lab of Metal Matrix Composites, School of Materials Science and Engineering, Shanghai Key Laboratory for Molecular Engineering of Chiral Drugs, Shanghai Jiao Tong University, 800 Dongchuan Road, Shanghai 200230, China; beilei1010@sjtu.edu.cn (B.W.); sravanbaddi@sjtu.edu.cn (S.B.); fengligao123@sjtu.edu.cn (F.G.); zcl@sjtu.edu.cn (C.Z.)

* Correspondence: douxiaoqiu@sjtu.edu.cn (X.D.); clfeng@sjtu.edu.cn (C.F.)

Citation: Lastname, F.; Lastname, F.; Lastname, F. Title. Journal Not Specified 2024, 1, 0. https://doi.org/.

Received:
Revised:
Accepted:
Published:

Copyright: © 2024 by the authors. Submitted to Journal Not Specified for possible open access publication under the terms and conditions of the Creative Commons Attribution (CC BY) license (https:// creativecommons.org/licenses/by/ 4.0/).

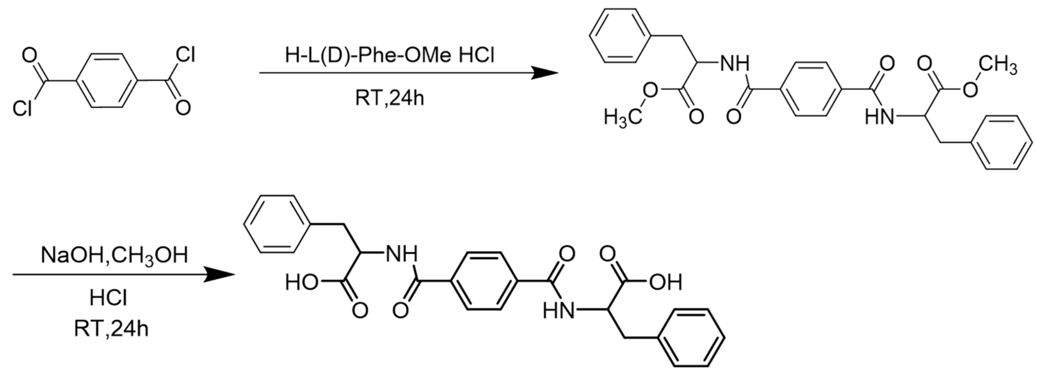

Figure S1. Synthesis procedures of L/DPF

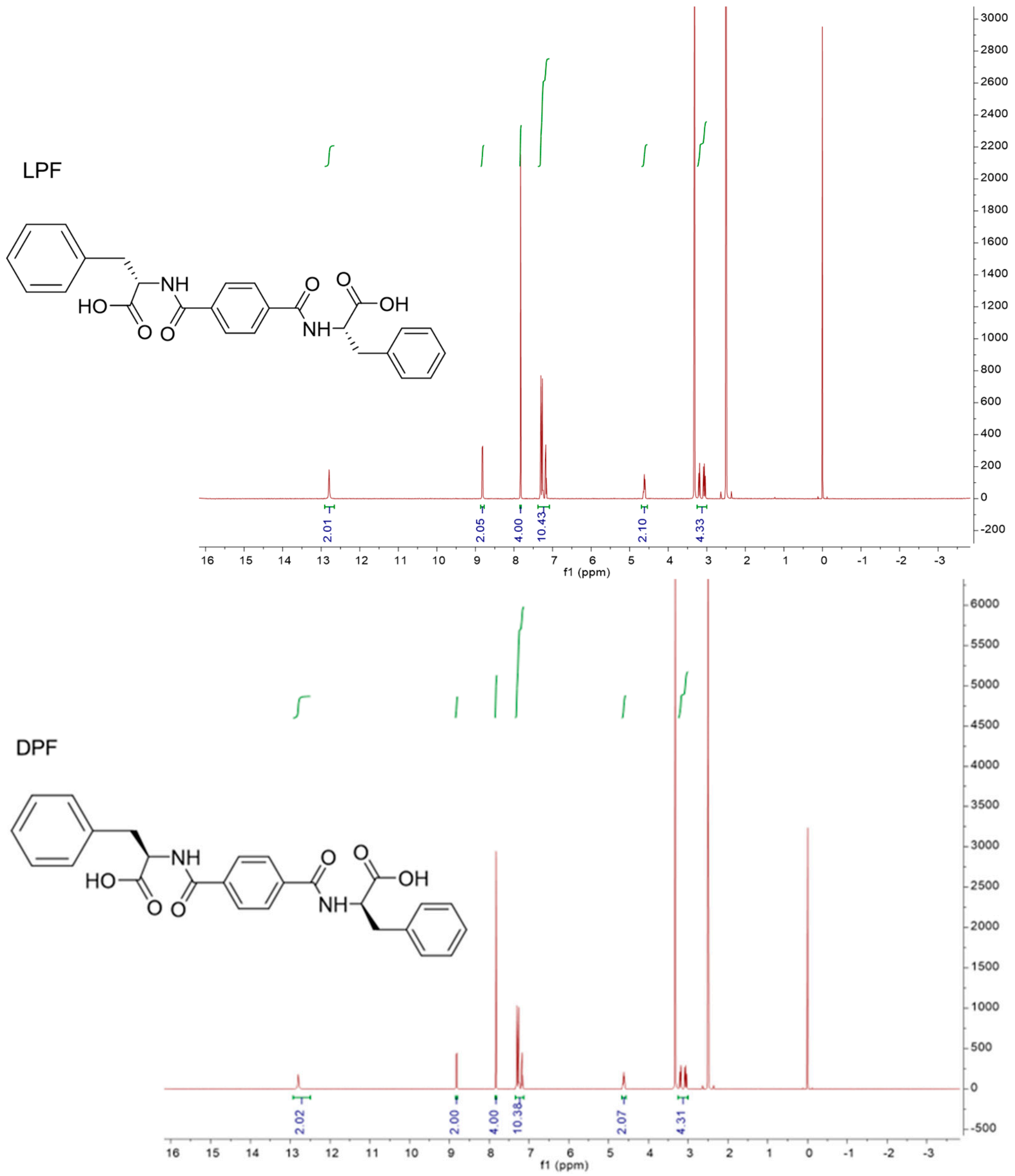

Figure S2. ^{1}H NMR (500 MHz) spectra of LPF and DPF in DMSO-d6.

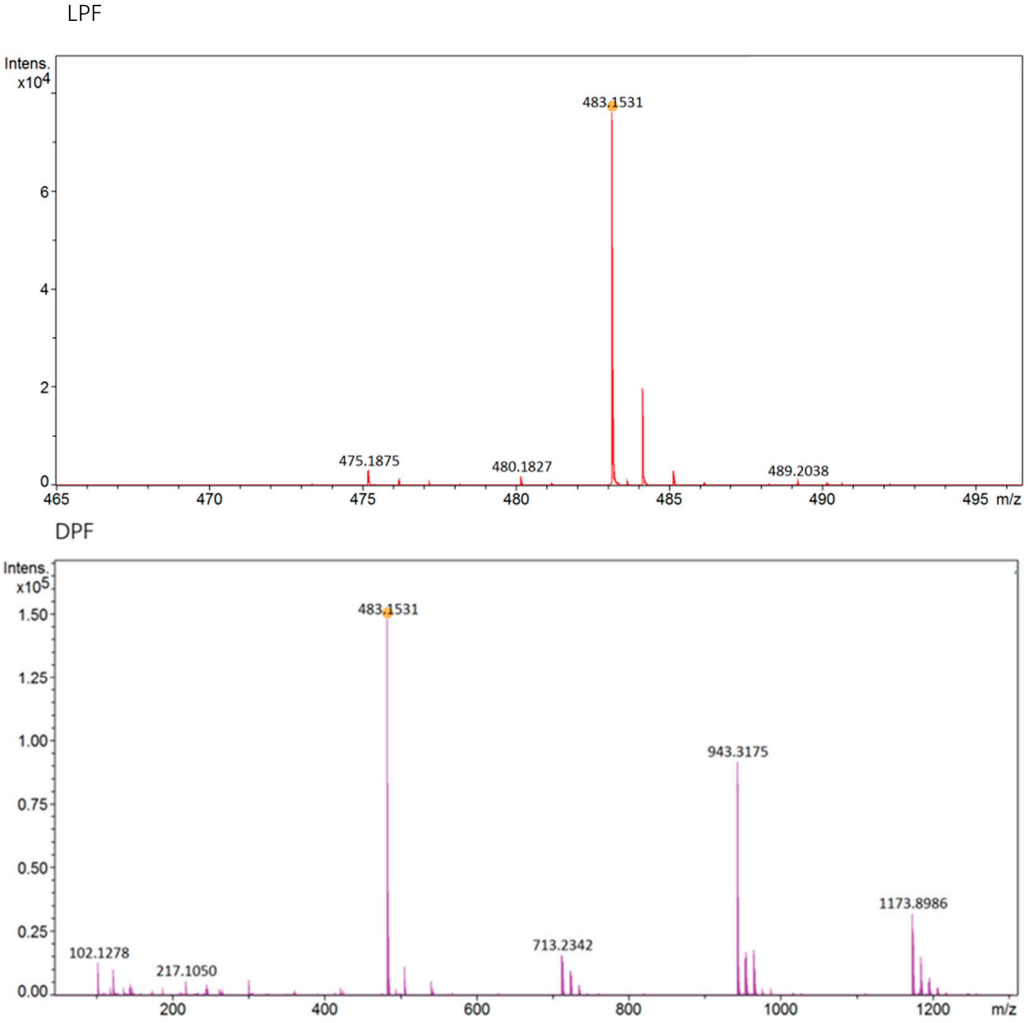

Figure S3. Mass spectrum of LPF and DPF.

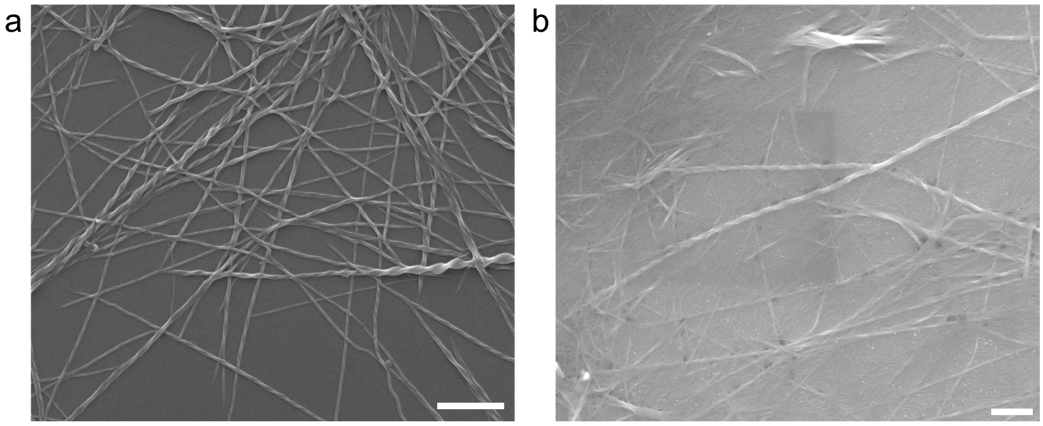

Figure S4. SEM images of **a** DPF_Mg hydrogel and **b** LPF_Mg hygrogel (scale bar = 1μm).

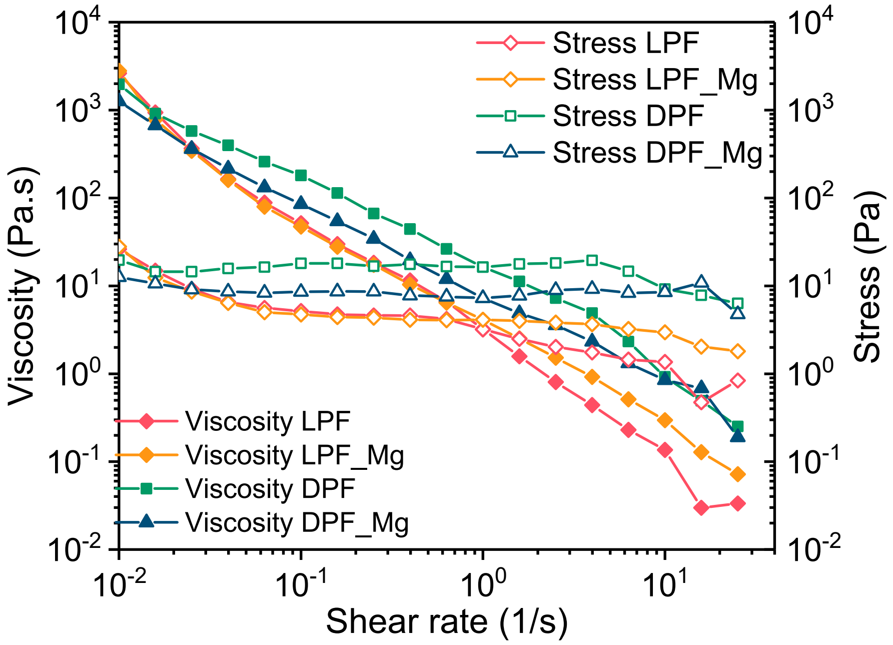

Figure S5. Rheological parameters for share rate VS. viscosity and share rate VS. share stress of L/DPF and L/DPF_Mg hydrogels.

Table 1. XPS survey recorded for LPF assemblies

Element	Position (eV)	Atom-%
C1s	284.79	79.15
O1s	531.98	15.74
N1s	399.72	5.11

Table 2. XPS survey recorded for LPF_Mg assemblies

Element	Position (eV)	Atom-%
C1s	284.8	79.98
O1s	532.13	16.19
N1s	399.74	4.32
Mg1s	1304.24	0.51

Table 3. XPS survey of C1s recorded for LPF assemblies

Functional group	Position (eV)	Atom-%
C-C	284.88	86.57
C-O-C	286.50	6.12
O-C=O	288.90	7.31

Table 4. XPS survey of C1s recorded for LPF_Mg assemblies

Functional group	Position (eV)	Atom-%
C-C	284.79	86.47
C-O-C	286.20	5.38
O-C=O	288.76	8.15

Table 5. XPS survey of O1s recorded for LPF assemblies

Functional group	Position (eV)	Atom-%
Metal carbonate	532.15	84.11
Metal hydroxide	533.50	15.89

Table 6. XPS survey of O1s recorded for LPF_Mg assemblies

Functional group	Position (eV)	Atom-%
Metal carbonate	532.16	95.48
Metal hydroxide	533.60	4.52

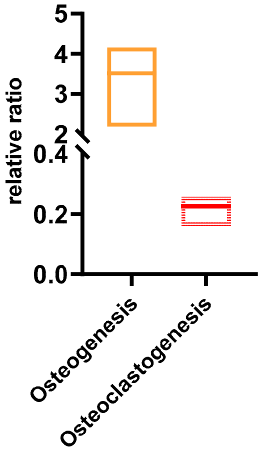

Figure S6. Relative ratio of osteogenesis and osteoclastogenesis for DPF_Mg group to the control group according to the positive rate from the ALP and Trap staining.

Table 7. Primers used in RT-qPCR experiments.

Gene Symbol	Forward primer	Reverse primer
RunX2	CCTGAACTCTGCACCAAGTCCT	TCATCTGGCTCAGATAGGAGGG
ALP	CCAGAAAGACACCTTGACTGTGG	TCTTGTCCGTGTCGCTCACCAT
OCN	GAACAGACAAGTCCCACACAGC	TCAGCAGAGTGAGCAGAAAGAT
OPN	CCGAGGTGATAGCTTGGCTT	ACAGGGATGACATCGAGGGA
TRAP	CTTGTTGACAGCGGTCCATCT	GGCTGAGAATTCGCCTGAGT
CTSK	CACTGACGAGATGGCACACT	TGTGGAGAATCGAACGGCAA
Atp6i	CTCATCAGGACCAACCGCTTCA	CGCCAAACATCACAGCGAAGAG
Nfatc1	AGGACCCGGAGTTCGACTT	AGGTGACACTAGGGGACACA
GAPDH	TGCCCCCATGTTTGTGATG	TGTGGTCATGAGCCCTTCC
